# Antiproliferative Effects of Cannabinoids and Cisplatin in Cervical Cancer Cells

**DOI:** 10.1002/cnr2.70561

**Published:** 2026-04-29

**Authors:** S. P. Mathibela, M. T. Lebelo, V. Steenkamp

**Affiliations:** ^1^ Department of Physiology, Faculty of Health Sciences University of Pretoria Pretoria South Africa; ^2^ Department of Pharmacology, Faculty of Health Sciences University of Pretoria Pretoria South Africa

**Keywords:** apoptosis, autophagy, cannabinoids, cervical cancer, cisplatin, combination therapy

## Abstract

**Introduction:**

Cervical cancer remains a leading cause of cancer‐related mortality among women globally, particularly in low‐ and middle‐income countries. Cisplatin, a standard chemotherapeutic agent, is limited by severe toxicities and chemoresistance. This study aimed to assess the effects of cisplatin in combination with phytocannabinoids, Δ9‐tetrahydrocannabinol (THC) and cannabidiol (CBD) on cell proliferation, morphology, cell cycle progression, cell death, and DNA damage.

**Methods:**

Synergistic interactions between THC, CBD, and cisplatin were assessed in HeLa, SiHa, and MCF‐12A cells using the checkerboard assay and SRB assay. Cell morphology, cell cycle progression, apoptosis induction, autophagic activity, and DNA repair gene expression were evaluated using various techniques.

**Results:**

The THC–CBD–cisplatin combination exhibited the strongest apoptotic response in cancer cells (HeLa 53%, SiHa 58%), while minimally affecting MCF‐12A cells (32%). Cannabinoid co‐treatment amplified the antiproliferative and pro‐apoptotic effects of cisplatin in HeLa and SiHa cells. The triple combination induced a G2/M arrest in HeLa cells and sub‐G1 accumulation in SiHa cells. Autophagic activity, indicated by LC3B puncta formation, increased in HeLa and SiHa cells following THC and CBD exposure. DNA repair genes *XRCC1* and *RAD51* were downregulated by the cannabinoid–cisplatin combination.

**Conclusion:**

These findings demonstrate that combining THC and CBD with cisplatin results in enhanced and mechanistically diverse anticancer effects, with a higher degree of selectivity for cervical cancer cells compared to non‐cancerous MCF‐12A cells by inducing apoptosis and autophagy while inhibiting DNA repair capacity. This study highlights the potential of cannabinoid‐based combination therapies as a promising approach for cervical cancer treatment.

## 1 Introduction

Cervical cancer is a leading cause of cancer‐related mortality among women globally, particularly in low‐ and middle‐income countries, where access to screening and treatment is limited [1]. Although treatment advances have progressively improved patient survival rates and enabled better detection and clinical management of the disease, cervical cancer remains the fourth most prevalent cause of cancer‐related deaths among women worldwide [2].

The standard therapeutic protocol typically involves a combination of surgical intervention and chemotherapy [2]. Conventional chemotherapy primarily employs platinum‐based agents, such as cisplatin [3]. Cisplatin targets the N7 reactive center on purine residues, causing DNA damage in cancer cells, which inhibits cell division and ultimately leads to apoptotic cell death [4]. Among the most significant DNA alterations are the 1,2‐intrastrand cross‐links of purine bases with cisplatin [4]. However, the effectiveness of these treatments is often constrained by high rates of chemoresistance and significant adverse effects. The most common and severe toxicity is nephrotoxicity, mainly due to damage to the proximal tubules of the kidneys [5]. Cisplatin accumulates in renal tubular epithelial cells, resulting in oxidative stress, inflammation, and apoptosis [6]. If left unaddressed, this can lead to acute kidney injury or chronic renal dysfunction [6]. Current cancer research is increasingly focused on enhancing the therapeutic index of cisplatin to improve its antitumor efficacy while reducing associated toxicities. These strategies include drug delivery innovations, rational combination therapies, biomarker integration, and personalized medicine approaches [7]. A promising strategy involves the use of cannabinoids as adjuncts to mitigate chemoresistance and the side effects associated with cisplatin.

Cannabinoids, a diverse group of bioactive compounds from the cannabis plant, have been shown to inhibit cancer cell proliferation through mechanisms such as inducing apoptosis, arresting the cell cycle, and inhibiting angiogenesis [8]. The two most prevalent cannabinoids, tetrahydrocannabinol (THC) and cannabidiol (CBD), have demonstrated potential in cancer research [8]. Studies indicate that cannabinoids exert their anticancer effects through various signaling mechanisms at multiple stages of tumor progression [9]. These mechanisms encompass the inhibition of cancer cell proliferation, metastasis, angiogenesis, and chemoresistance, along with the induction of apoptosis and autophagy [9]. Numerous in vivo studies have highlighted the effects of THC and CBD on different cancer types, including glioma, myeloma, and leukemia [9]. Furthermore, when cannabinoids are combined with conventional agents such as cisplatin, they exhibit the potential to enhance anticancer effects and to overcome drug resistance [10]. The synergistic interactions between cannabinoids and cisplatin can augment treatment efficacy by simultaneously targeting multiple cellular pathways [10]. For instance, the combination of THC and CBD with vinblastine in leukemia cells enhanced the cytotoxic effect of vinblastine in resistant leukemia cells via the downregulation of P‐glycoprotein [10]. These combinatorial strategies aim to reduce the dosage and side effects of chemotherapeutic drugs and to achieve more comprehensive and durable anticancer responses.

Considering cisplatin's crucial role in cervical cancer chemotherapy and its well‐established nephrotoxicity, alongside less common but context‐ dependent cardiotoxicity [11], with cannabidiol's anticancer and cardioprotective properties [12] and THC's therapeutic effectiveness for issues like chemotherapy‐induced nausea [13], this integrative approach highlights the potential of using cannabinoids together with conventional therapies to improve cervical cancer treatment outcomes. Therefore, this study aimed to investigate the anticancer effects of combining cannabinoids with cisplatin in cervical cancer cells.

## 2 Methods and Materials

### 2.1 Test Compounds

The cannabinoids THC and CBD were sourced from LECO Africa (Pty) Ltd. (South Africa), while cisplatin was obtained from Sigma‐Aldrich (St. Louis, USA). The CBD stock solution, at a concentration of 3.225 mM, was dissolved in methanol and then diluted with the appropriate cell culture media to create a 20 μM working solution. This was further diluted to achieve test concentrations ranging from 0.039 to 10 μM. Similarly, the THC stock solution, with a concentration of 3.184 mM, was dissolved in 1 mL of methanol and diluted in the appropriate cell culture media to produce a 60 μM working solution. This solution was further diluted to obtain a concentration range of 0.234–30 μM for use in experimental procedures. The cisplatin stock solution, prepared at 20 mM in dimethyl sulfoxide (DMSO), was diluted to 200 μM with the appropriate cell culture media and further diluted to achieve a concentration range of 0.39–100 μM for the assays. Actinomycin D, used as a positive control, was prepared as a stock solution at 1 mg/mL in DMSO and further diluted to the required concentrations for the assays.

### 2.2 Cell Culture

The HeLa cervical cancer cell line was procured from the American Type Culture Collection (Virginia, USA). The SiHa cervical cancer cell line was gifted by the Pan African Cancer Research Institute (University of Pretoria, South Africa). The MCF‐12A non‐cancerous breast cell line was bought from Highveld Biological (Johannesburg, South Africa). Both cervical cancer cell lines were grown in 75 cm^2^ flasks in Dulbecco's Modified Eagle Medium (DMEM), to which was added 10% fetal calf serum (FCS) and 1% penicillin–streptomycin. Flasks were kept in an incubator with 5% CO_2_ at 37°C. The MCF‐12A cells were grown in a similar way, but their medium was a combination of 1:1 DMEM and Hams F12. The latter contained epidermal growth factor, cholera toxin, insulin, and hydrocortisone, along with 10% heat‐inactivated FCS, penicillin, streptomycin, and fungizone. The cells were grown until 80% confluence, after which flasks were washed with phosphate buffered saline (PBS), and the cells detached using trypsin/versene. The cells were centrifuged and counted using the trypan blue test. Thereafter, the cells were mixed with DMEM to the concentration required for the tests. The cell lines used in this study underwent periodic testing for mycoplasma to ensure that they were free of mycoplasma contamination.

### 2.3 Cytotoxicity

Cytotoxicity of the test compounds was assessed using the sulforhodamine B (SRB) staining assay as described by Vichai and Kirtikara [14], with minor changes to volumes used. The HeLa, SiHa, and MCF‐12A cells were seeded in 96‐well plates at a density of 5000 cells per well and incubated for 24 h at 37°C and 5% CO_2_ to allow for attachment. Each plate contained three sets of controls: negative control (NC; cells propagated in growth medium), the vehicle control (VC; growth media containing 0.01% methanol), and the positive control (30 μM Cisplatin). DMEM was supplemented with 0.5% FCS in the assays. The cells were treated with varying concentrations of CBD (0.039–10 μM) and THC (0.234–30 μM) for a period of 24, 48, and 72 h. Each drug was tested individually using separate plates. Following the exposure period, 50 μL of a 50% trichloroacetic acid (TCA; Sigma‐Aldrich, USA) solution was added to each well for fixation of the cells, and the plates were incubated at 4°C for 24 h. Plates were washed three times with slow‐running tap water and dried in an oven (EcoTherm, Labotec, South Africa) set at 40°C to 45°C. A volume of 100 μL of 0.057% SRB solution (Sigma‐Aldrich, St. Louis, USA) was added to each well, and the plates were incubated at room temperature for 30 min in the dark. Thereafter, unbound dye was removed by washing the plates three times with 150 μL of 1% acetic acid solution (Sigma‐Aldrich, St. Louis, USA) and dried in the oven. The bound dye was extracted from fixed cells by adding 200 μL of 10 mM Tris‐base solution (Sigma‐Aldrich, St. Louis, USA) to each well. Plates were placed on a gyratory shaker (VRN‐200, Gemmy Industrial Corporation, Taiwan) for 1 h to solubilize the protein‐bound dye. After the dye had solubilized, the optical density (OD) was measured at 540 nm (reference: 630 nm) using an ELX 800 microplate reader (BioTek instruments Inc., Highland Park, USA). All values were blank‐subtracted, and the cell density (%) calculated using the following formula:

$$\begin{array}{c} \text{Cell density}\;\left(\%\;\text{relative to negative control}\right)=\\ {\mathrm{OD}}_{\text{sample}}/\text{Average}\;{\mathrm{OD}}_{\text{negative}}\times 100 \end{array}$$

where “OD_sample_” refers to the corrected optical density of the sample and “OD_negative_” is the corrected optical density of the negative control.

The optimal incubation period was 72 h, which was used for subsequent experiments.

### 2.4 Assessment of Synergism

Synergistic interactions between THC, CBD, and cisplatin were assessed in HeLa, SiHa, and MCF‐12A cells using the checkerboard assay [15]. The assay was conducted by seeding 5000 cells per well in 96‐well plates, with a final volume of 100 μL per well. CBD concentrations ranged from 0–2 μM, THC concentrations from 0–5.2 μM, and cisplatin concentrations from 0–150.52 μM. The SRB assay, as described above, was used to assess cell density after 72 h treatment. Analysis was performed using SynergyFinder 2.0 and the Bliss Independence drug interaction model. A score of < −10 was considered antagonistic, between −10 and 10 as additive, and > 10 as synergistic activity. Drug combination responses were plotted as concentration‐response curves using GraphPad Prism software version 8.0 (California, USA) to determine the statistical significance (*p* < 0.05) of synergistic combinations.

### 2.5 Light Microscopy

#### 2.5.1 Polarization‐Optical Transmitted Light Differential Interference Contrast

The HeLa, SiHa, and MCF‐12A cells were seeded into six‐well plates (Sigma‐Aldrich, USA) at a density of 500 000 cells per well and incubated at 37°C and 5% CO_2_ for 24 h to allow for the attachment of cells to the plate. The cells were treated with the following half‐maximal inhibitory concentration (IC_50_): for HeLa cells—2.34 μM CBD, 2.30 μM THC, and 6.60 μM cisplatin; for SiHa cells—0.50 μM CBD, 1.30 μM THC, and 37.63 μM cisplatin; and for MCF‐12A cells—8.20 μM CBD, 28 μM THC, and 28 μM cisplatin. For combination treatments, HeLa cells were exposed to 1.15 μM THC + 1.17 μM CBD; 2.3 μM THC + 1.65 μM cisplatin; 0.58 μM CBD + 6.6 μM cisplatin; and 1.15 μM THC + 1.17 μM CBD + 3.3 μM cisplatin. SiHa cells were treated with 0.65 μM THC + 0.25 μM CBD; 0.65 μM THC + 18.82 μM cisplatin; 0.25 μM CBD + 18.82 μM cisplatin; and 0.65 μM THC + 0.25 μM CBD + 9.41 μM cisplatin. For MCF‐12A cells, the following treatment combinations were used: 14 μM THC + 4.1 μM CBD; 14 μM THC + 28 μM cisplatin; 8.2 μM CBD + 7 μM cisplatin; and 14 μM THC + 32.8 μM CBD + 14 μM cisplatin.

An Axiovert 40 CFL microscope (Zeiss, Oberkochen, Germany) was used to capture images at 40X magnification. The morphology of 100 cells was examined using Carl Zeiss TM AxioVision Rel. 4.8 software (Zeiss, Oberkochen, Germany), where the number of cells demonstrating aberrant and normal morphology was recorded. Image J software (National Institutes of Health, Bethesda, Maryland, USA) was used to count the cells. The data obtained was analyzed using Microsoft Excel 2016 (Redmond, Washington, USA), and GraphPad software version 8 (GraphPad Software Inc., California, USA) was used to plot the graphs.

#### 2.5.2 Hematoxylin and Eosin Staining

HeLa, SiHa, and MCF‐12A cells were seeded into six‐well plates containing heat‐sterilized coverslips (Sigma‐Aldrich, USA) at a density of 500 000 cells per well in 3 mL of medium. Cells were exposed to the different treatments as detailed in Section 2.5.1 for 72 h. Following incubation, the coverslips were removed from the wells and fixed with Bouin's fixative for 30 min. The fixative was discarded, and the coverslips were immersed in 70% ethanol for 20 min before rinsing them with tap water. Meyer's hematoxylin was added, and the coverslips were allowed to stand for 20 min before rinsing with tap water and ethanol (70%). Subsequently, 1% eosin was used to stain the cells for 5 min. Cover slips were rinsed twice sequentially with 70%, 96%, and 100% ethanol and then xylene to ensure that excess water was removed. The coverslips were then left to dry, after which they were viewed under a Carl Zeiss TM AxioVision microscope with Rel. 4.8 software (Zeiss, Oberkochen, Germany). Data capturing consisted of qualitative image examination, as well as quantitative mitotic indices. The latter was acquired by analyzing and counting mitosis phases (prophase, metaphase, anaphase and telophase) of 1000 cells per repeat using Image J software (National Institutes of Health, Bethesda, Maryland, USA).

### 2.6 Flow Cytometry

#### 2.6.1 Cell Cycle Progression

HeLa and SiHa cells were seeded at 750 000 cells per T25 cm^2^ flask and left for 24 h to allow for attachment in an incubator with humidified atmosphere at 37°C and 5% CO_2_. Cells were exposed to the conditions as described in Section 2.5.1 for 72 h. After the incubation period, the cells were trypsinized and re‐suspended in 1 mL PBS and centrifuged for 5 min at 300 × *g*. The supernatant was removed and discarded, and cells were re‐suspended in ice cold PBS (200 μL) containing 0.1% FCS. Thereafter, ice cold ethanol (70%, 4 mL) was added to the cells in a drop wise manner on a vortex, and the samples were kept at 4°C for 24 h. Subsequently, cells were centrifuged at 300 × *g* for 5 min and the supernatant was removed. Cells were then re‐suspended in 1 mL PBS containing propidium iodide (PI, 40 μg/mL), RNAse A (100 μg/mL), and triton X‐100 (0.1%), and samples were incubated at 37°C for 45 min. PI fluorescence was measured using a Cytoflex flow cytometer (Beckman Coulter Inc. (Brea, California, USA)). A total of 10 000 events were recorded per treatment. Cell cycle distributions were calculated using the Kaluza analysis software (Beckman Coulter Inc. (Brea, California, USA)) by assigning relative DNA content per cell to sub‐G 1‐, G1‐, S‐ and G2/M fractions. The data obtained were further analyzed using Microsoft Excel 2016 (Redmond, Washington, USA).

##### 2.6.1.1 Apoptosis

The Annexin V/PI assay was performed according to the manufacturer's protocol (Biocom Biotech, Centurion, South Africa) [16]. The HeLa, SiHa, and MCF‐12A cells were seeded at a density of 500 000 cells per 25 cm^2^ tissue culture flask and incubated at 37°C and 5% CO_2_ for 24 h to allow for attachment. Cells were exposed to the compounds as described in Section 2.5.1 for 72 h. Thereafter, cells were trypsinized, centrifuged at 300 × *g* for 5 min, and the supernatant discarded. The pellet was re‐suspended in 1 mL 1× PBS and centrifuged for 5 min at 300 × *g*. The supernatant was discarded, and the pellet was re‐suspended with 1× binding buffer. The supernatant was discarded, and the pellet was re‐suspended in 500 μL 1× Annexin binding buffer followed by 5 μL Annexin V and 5 μL PI and vortexed prior to 15 min incubation in the dark. The samples were analyzed using a Cytoflex flow cytometer (Beckman Coulter Inc. (Brea, California, USA)). Analysis of data was done using the Kaluza analysis software from Beckman Coulter Inc. (Brea, California, USA).

### 2.7 Autophagic Activity

The HeLa, SiHa, and MCF‐12A cells were seeded into six‐well plates at a density of 500 000 cells/3 mL medium/well containing heat‐sterilized coverslips (Sigma‐Aldrich, USA). The cells were incubated overnight at 37°C to allow for attachment to the coverslips. Cells were exposed as described in Section 2.5.1 for 72 h. Cells were fixed by incubating slides for 15 min in 1 mL 2% paraformaldehyde at room temperature. Coverslips were then washed with 1× PBS (3 times) and incubated for 5 min in 1 mL 0.2% Triton‐X to permeabilize the cell membranes. Cells were washed three times in 1× PBS and blocked overnight in 1 mL 2% bovine serum albumin (BSA). Coverslips were incubated in LC3B primary antibody (1:200 dilution in PBS) with 2% BSA for 1 h at room temperature (RT). After washing the coverslips with PBS three times, slides were incubated with 30 μL secondary Ab conjugated with Alexa Fluor 488 secondary antibody (1:200 dilution in 2% BSA) for 1 h at RT (in the dark). Coverslips were then mounted on microscope slides using Fluoromount mounting fluid containing 4′,6‐diamidino‐2‐phenylindole (DAPI) and allowed to dry at RT in the dark for 24 h. Slides were preserved at 4°C. A Zeiss LSM800 confocal microscope was used to visualize the slides using a 40× oil objective. The ZEN blue software (ZEISS) was set to capture 16 bits per pixel at a pixel dwell time of 9.84 μsec and the optimal frame size (1716 px by 1716 px) was used. The averaging was set to 4 and the pinhole to 1 AU. Laser power did not exceed 2% and gain was adjusted as needed between 700 and 800 V.

### 2.8 Gene Expression

The HeLa, SiHa, and MCF‐12A cells were seeded at a density of 10 000 cells per well into a 12‐well plate (Sigma‐Aldrich, USA). Cells were exposed to the test compounds as mentioned in Section 2.5.1 for 72 h. The mRNA was extracted using the ISOLATE II RNA Mini Kit (Bioline Meridian Bioscience, UK). A lysis buffer was added to lyse cells. Thereafter, the lysate was vortexed, filtered, and centrifuged (11000 × *g*) for 1 min at 4°C. Ethanol (70%) was used to homogenize the lysate. The lysate was then loaded into the ISOLATE II RNA mini column and centrifuged for 30 s at 11000 × *g*. The membrane desalting buffer was added to desalt the silica membrane. DNAse I reaction mixture was directly applied to the silica membrane to digest the DNA. The silica membrane was incubated for 15 min at room temperature. Dilution of 10% of the reconstituted DNase I reaction mixture was used to prepare the reaction buffer DNase. The silica membrane was cleaned with Wash Buffer before being dried. The eluted RNA was kept on ice. RNA was reverse transcribed using M‐MuLV reverse transcriptase per the manufacturer's instructions (ELK Biotechnology, USA). The RNA then underwent oven incubation at 42°C for 1 h. The enzyme was denatured at 90°C and the complementary DNA (cDNA) was stored at −80°C until analysis. The primers that were used for the DNA repair genes were *XRCC1* and *RAD51*, whereas glyceraldehyde 3‐phosphate dehydrogenase (*GAPDH*) was used as a housekeeping gene (Table 1). The cDNA was added to both the forward and reverse primers. The samples were centrifuged before being placed in a light cycler for three‐step cycling (Table 2). The analysis was carried out using the 2^−ΔΔCT^ method. The data was normalized with glyceraldehyde‐3‐phosphate dehydrogenase (*GAPDH*) before being analyzed using Microsoft Excel 2010 (Microsoft Corporation, Washington, USA). Graph‐Pad software version 10.1 (Graphpad Software Inc., California, USA) was used to plot the graphs.

**TABLE 1:** Primers used in polymerase chain reaction to assess DNA damage and repair mechanisms.

Gene	Forward primer sequence (5′‐3′)	Backward primer sequence (3′‐5′)
*GAPDH*	GAAGCCTGCCGGTGACTAA	GCCCAATACGACCAAATCAGAG
*XRCC1*	CCCCAGTGGTGCTAACCTAAT	ACTGGGGCTGTGGTGGGGTA
*RAD51*	GCTGGGAACTGCAACTCATCT	GCAGCGTCCTCTCTCCAGC

**TABLE 2:** The three‐step polymerase chain reaction cycling process.

Cycles	Temperature	Duration	Step
1	94	3 min	Polymerase activation
2	94	30 s	Denaturing
	55	60 s	Annealing
	72	60 s	Extension
3	72	5 min	Final Extension

### 2.9 Statistical Analysis

Statistical analysis was performed using GraphPad Prism software version 8.0 (California, USA). The data is presented as the mean ± SD of at least three assays carried out in triplicate. Comparisons of three means were performed by a *t*‐test, and comparisons of multiple means were analyzed by an ANOVA. A *p* < 0.05 was considered statistically significant.

## 3 Results

### 3.1 Synergism

**FIGURE 1:**
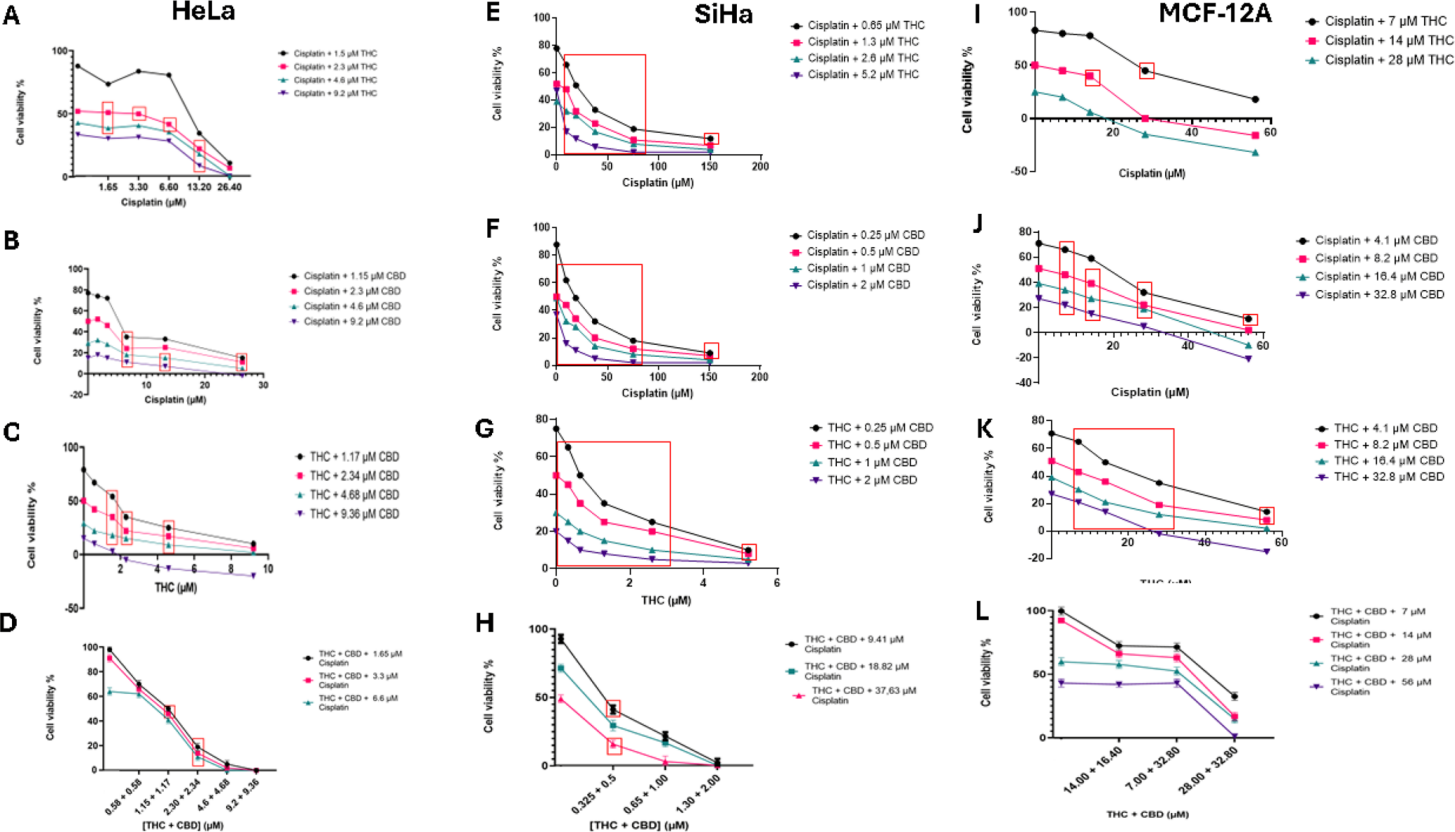
Assessment of synergy between THC, CBD, and cisplatin. Concentration response curves of cisplatin combined with THC or CBD and THC combined with CBD in HeLa cells (A–D), SiHa cells (E–H), and MCF‐12A cells (I–L). Red box represents concentration combinations that are synergistic as determined by the bliss independence model using SynergyFinder 2.0 software and that are significantly different from THC‐CBD‐cisplatin treatment alone.

Synergistic effects were observed in multiple drug combinations across HeLa and SiHa cancer cell lines, including THC with cisplatin, CBD with cisplatin, CBD with THC, and the triple combination of THC, CBD, and cisplatin (Figure 1A–H). In the MCF‐12A, non‐cancerous cells, synergy was observed between THC and cisplatin, CBD and cisplatin, and CBD and THC, with the THC‐CBD combination showing the most significant synergy (Figure 1I–K). Interestingly, an antagonistic effect was observed with the THC‐CBD‐cisplatin combination in non‐cancerous cells (Figure 1L). Synergistic effects were consistent in both cancer cell lines, while in the non‐cancerous MCF‐12A cells, synergy was observed for most combinations except for the THC‐CBD‐cisplatin combination (Figure 1L). A summary of the drug combinations across the cell lines is provided in Table 3.

**TABLE 3:** Summary of drug combination interactions across HeLa, SiHa, and MCF‐12A cell lines.

Drug combination	HeLa cells	SiHa cells	MCF‐12A cells
THC + Cisplatin	Synergistic	Synergistic	Synergistic
CBD + Cisplatin	Synergistic	Synergistic	Synergistic
THC + CBD	Synergistic	Synergistic	Synergistic
THC + CBD + Cisplatin	Synergistic	Synergistic	Antagonistic

### 3.2 Morphological Features of Cell Death

**FIGURE 2:**
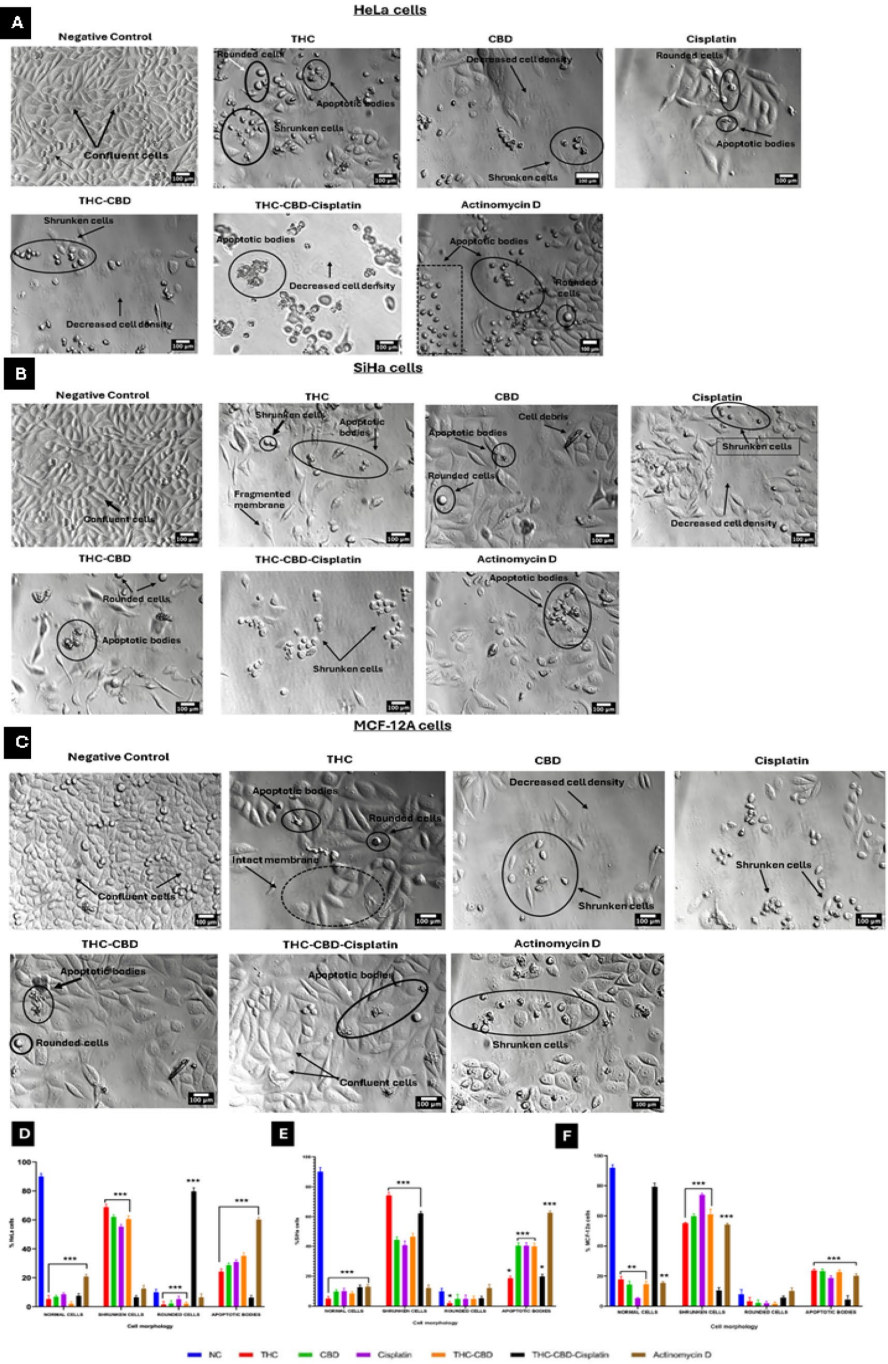
Micrographs of (A) HeLa, (B) SiHa, and (C) MCF‐12A cells after 72 h of treatment. Scale bar = 100 μm. Bar graphs (D–F) indicate quantification of morphological features in HeLa, SiHa and MCF‐12A cells, respectively. Standard deviation represented by T‐bars; **p < 0.05; **p < 0.01; and ***p < 0.001 indicates statistical difference between compounds and negative control (NC).

PlasDIC was utilized to assess cell morphology, following a 72 h exposure to the concentrations outlined in Section 2.5.1, on HeLa, SiHa, and MCF‐12A cells. Treatment of HeLa cells with THC, CBD, and cisplatin led to the appearance of shrunken cells and apoptotic bodies (Figure 2A). The combination of THC‐CBD‐cisplatin resulted in 6.5% shrunken cells and 6.3% apoptotic bodies (Figure 2D). A significant (*p* < 0.05) increase in rounded cells (79.7%) was observed (Figure 2D). SiHa cells (Figure 2B) exhibited a significant (*p* < 0.05) increase in shrunken cells and apoptotic bodies when treated with THC, CBD, cisplatin, and the THC‐CBD‐cisplatin combination (Figure 2E). Similarly, MCF‐12A cells (Figure 2C) showed a significant (*p* < 0.05) increase in shrunken cells and apoptotic bodies under the same treatment conditions. There was no significant decrease in normal cells when treated with the THC‐CBD‐cisplatin combination (Figure 2F).

### 3.3 Mitotic Arrest

**FIGURE 3:**
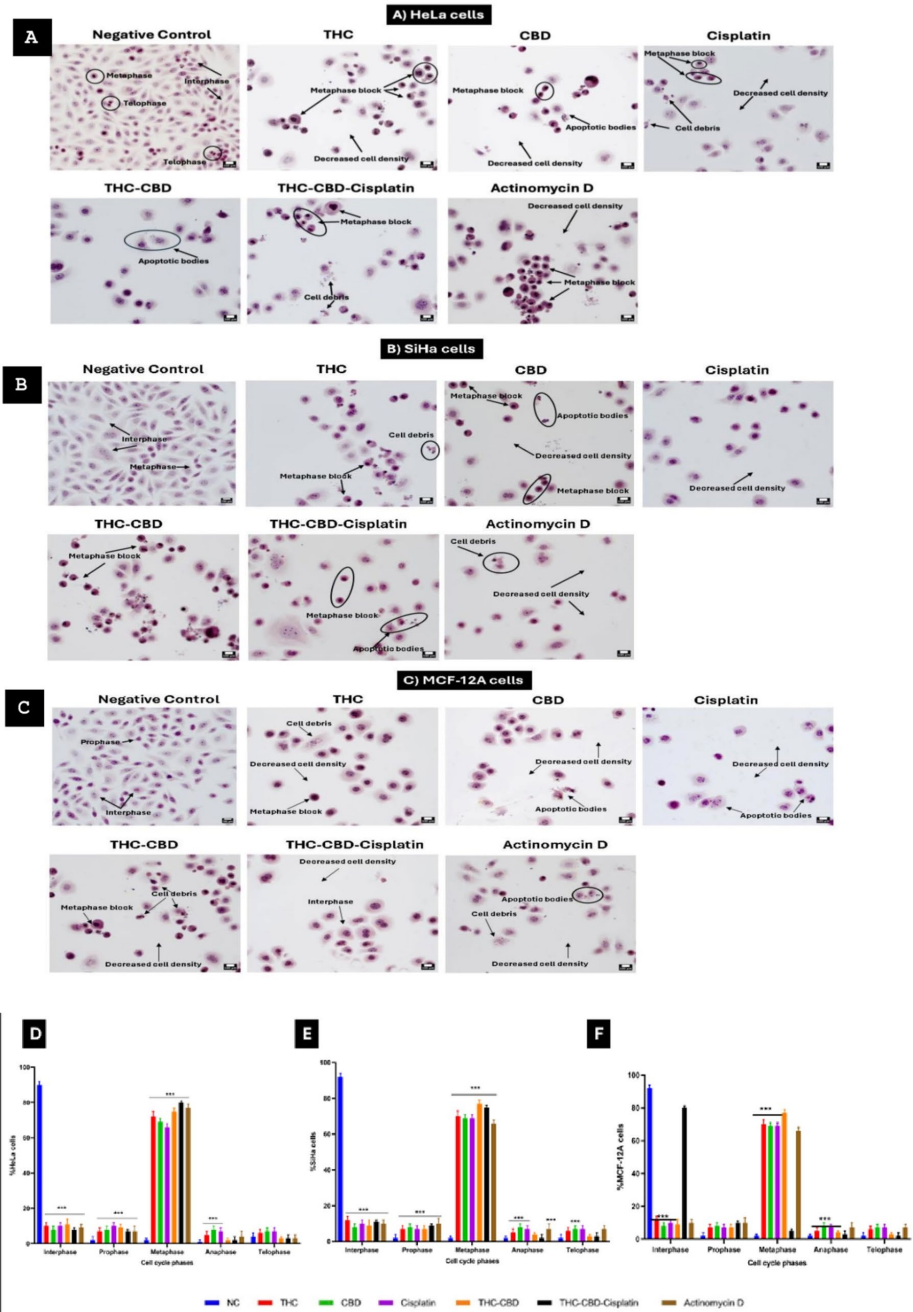
Hematoxylin and Eosin staining images of (A) HeLa, (B) SiHa, and (C) MCF‐12A cells after 72 h of treatment. Bar graphs (D–F) represent quantification of cell cycle phases in HeLa, SiHa and MCF‐12A cells, respectively. Standard deviation represented by T‐bars; ***p < 0.001 indicates statistical difference between compounds and negative control (NC).

In the negative control groups, the majority of cells were in interphase: HeLa cells 90%, SiHa cells 92%, and MCF‐12A cells 92%. Treatment with THC, CBD, or cisplatin individually led to an increased number of cells in metaphase across all three cell lines. When treated with the triple combination of THC‐CBD‐cisplatin, an increase in metaphase was noted in HeLa cells (80%, Figure 3A) and SiHa cells (75%, Figure 3B), while the non‐cancerous MCF‐12A cells showed more cells in interphase (80%, Figure 3C). Quantitation of the cell cycle phases after treatment with THC, CBD, cisplatin and their combinations in HeLa, SiHa and MCF‐12A cells is provided in Figure 3D–F.

### 3.4 Cell Cycle Arrest

**FIGURE 4:**
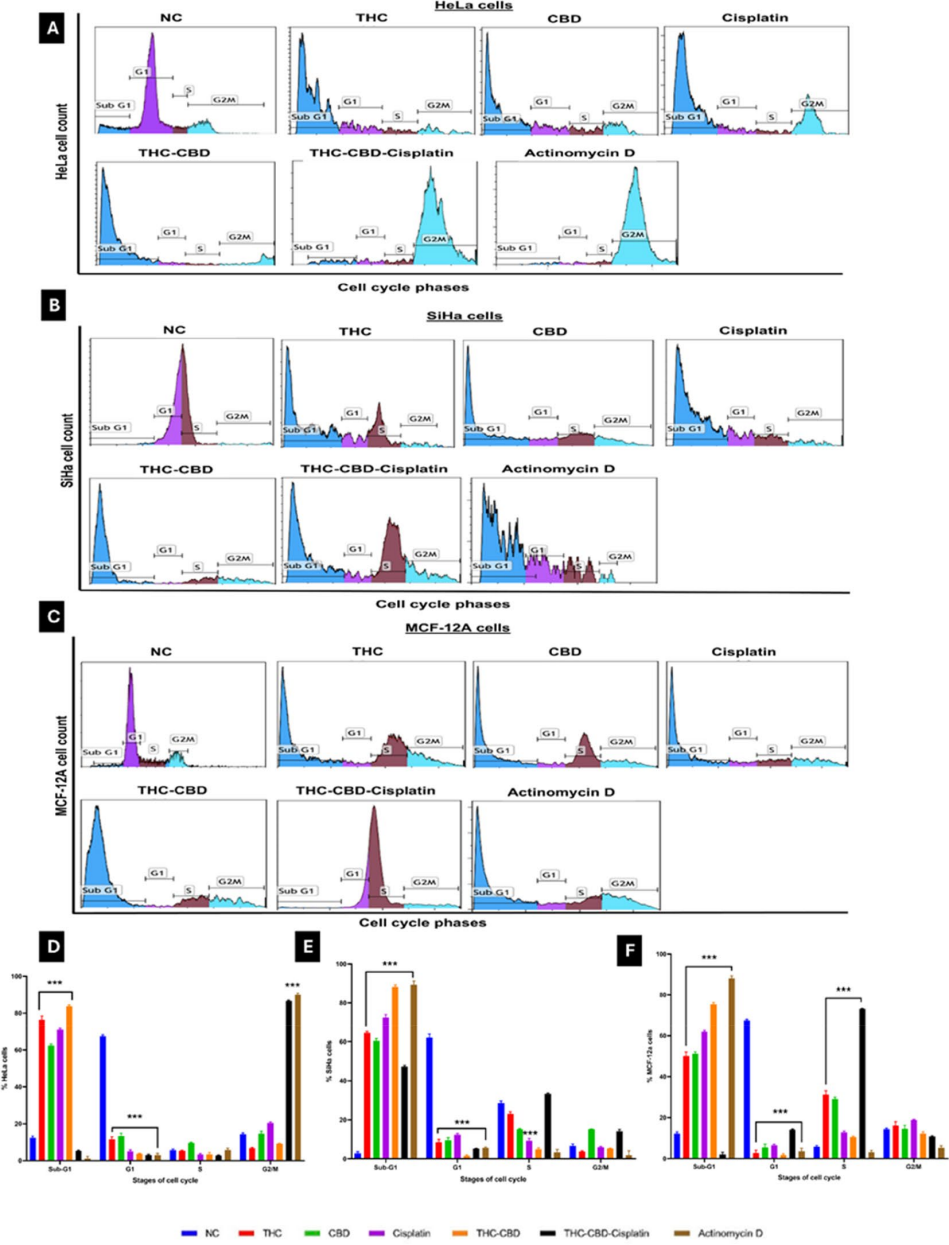
Cell cycle distribution of (A) HeLa cells, (B) SiHa cells, and (C) MCF‐12A cells after 72 h of treatment. Bar graphs (D–F) represent the quantification of the cell cycle analysis in Hela, SiHa and MCF‐12A cells, respectively. Standard deviation represented by T‐bars; ***p < 0.001 indicates statistical difference between compounds and negative control (NC).

The cell cycle distribution of HeLa cells exposed to media with 0.5% FCS as a negative control (Figure 4A) showed a relatively normal distribution after 72 h, with 12.4% in the sub‐G1 phase, 67.5% in the G1 phase, 5.6% in the S‐phase, and 14.4% in the G2/M phase. In contrast, SiHa cells (Figure 4B) exhibited 2.8% in the sub‐G1 phase, 62.1% in the G1 phase, 28.6% in the S‐phase, and 6.7% in the G2/M phase. For MCF‐12A cells (Figure 4C), 12.3% were in the sub‐G1 phase, 67.4% in the G1 phase, 5.8% in the S‐phase, and 14.4% in the G2/M phase. When HeLa cells were treated with the THC‐CBD‐cisplatin combination (Figure 4D), there was a notable increase in the G2/M phase (86.7%) and a significant decrease in the G1 phase (3.1%, *p* < 0.001). In SiHa cells, the THC‐CBD‐cisplatin combination (Figure 4E) led to a substantial increase in the sub‐G1 phase (47.3%) and an increase in the G2/M phase (14.2%). In MCF‐12A cells (Figure 4F), the combination treatment led to an increase in the S phase (73.1%) accompanied by a marked reduction in the G1 phase (14.1%).

### 3.5 Apoptosis Induction

**FIGURE 5:**
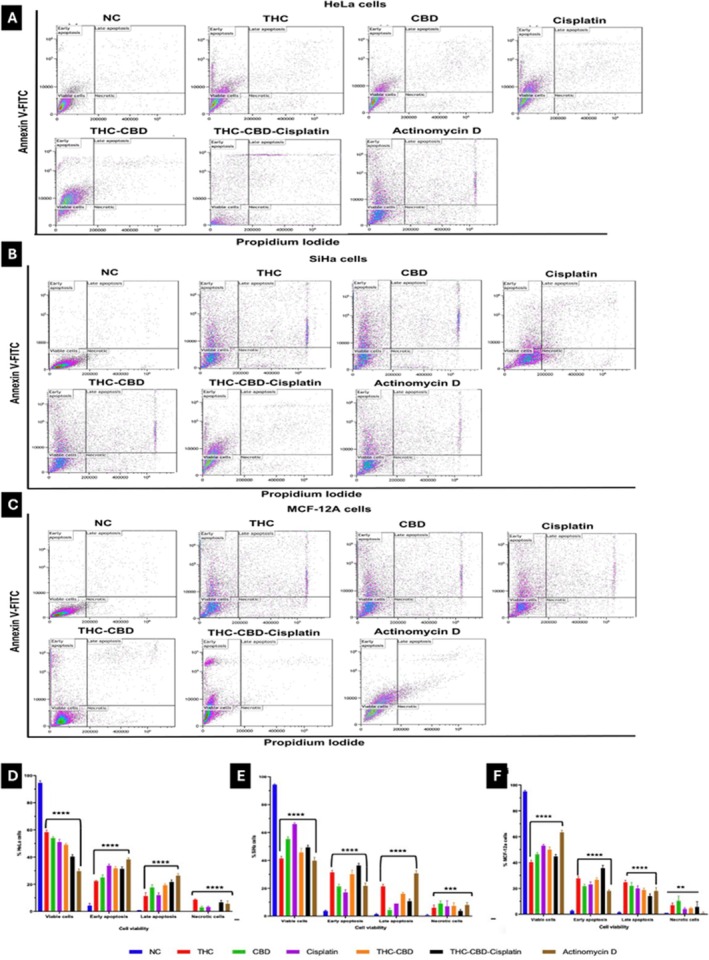
Annexin‐V FITC/PI flow cytometric analysis of (A) HeLa cells, (B) SiHa cells, and (C) MCF‐12A cells after 72 h of treatment. Bar graphs (D–F) represent the quantification of early and late apoptotic, necrotic, and viable cell populations in HeLa, SiHa, and MCF‐12A cells, respectively. Standard deviation represented by T‐bars; **p < 0.01 and ***p < 0.001 indicates statistical difference between compounds and negative control (NC).

Treatment of HeLa cells with individual drugs induced moderate levels of apoptosis (early and late stages), with THC being 34%, CBD 43%, and cisplatin 46% (Figure 5A). The combination of THC, CBD, and cisplatin further enhanced this effect, increasing apoptosis to 53% (Figure 5A). In SiHa cells (Figure 5B), THC alone produced the highest apoptosis rate among the single treatments (53%), which was further elevated to 58% with the THC–CBD–cisplatin combination, compared to 36% for CBD and 27% for cisplatin. In MCF‐12A non‐tumorigenic cells (Figure 5D), treatment with single agents induced moderate apoptosis (THC 52%, CBD 44%, and cisplatin 44%), while the THC–CBD–cisplatin combination produced a similar apoptotic level (53%), accompanied by a slight increase in necrosis. Overall, the THC–CBD–cisplatin combination elicited the most pronounced apoptotic response in cancer cells (HeLa 53%, SiHa 58%) (Figure 5D,E), whereas its effect on MCF‐12A cells was comparatively less selective (Figure 5F).

### 3.6 LC3B Puncta Formation

**FIGURE 6:**
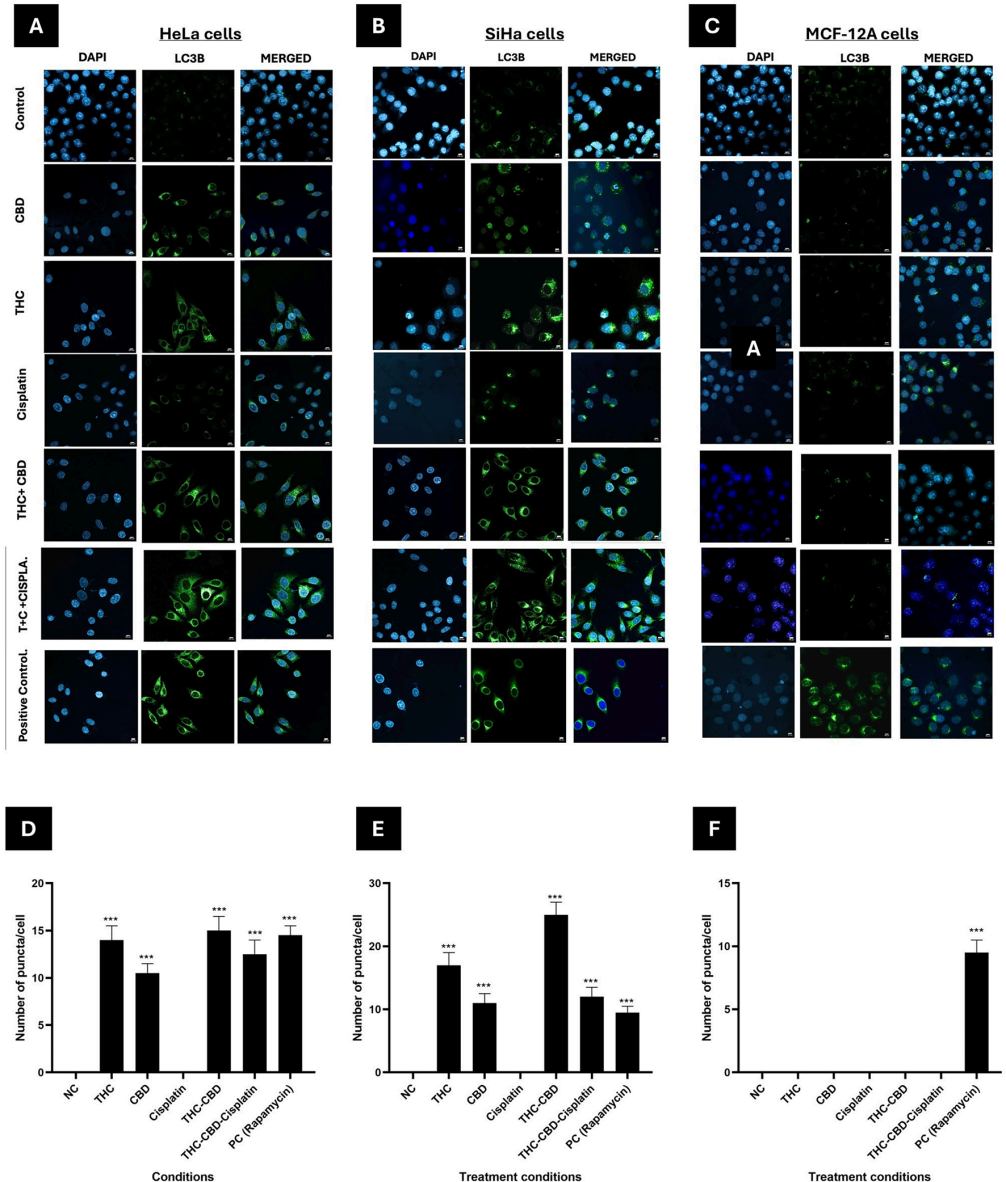
Immunofluorescence analysis of autophagic activity in HeLa, SiHa, and MCF‐12A cells after 72 h of treatment. Representative immunofluorescence images showing autophagic activity in (A) HeLa, (B) SiHa, and (C) MCF‐12A cells after 72 h of treatment. Cells were stained for LC3B (green), an indicator of autophagy, and the nuclei were counterstained with DAPI (blue). Bar graphs (D–F) represent the quantification of LC3B puncta per cell in HeLa, SiHa and MCF‐12A cells, resprectively. Standard deviation represented by T‐bars; ***p < 0.001. NC, negative control; PC, positive control (Rapamycin, 0.5 μM).

Cells exposed to media containing 0.5% FCS as a negative control showed no LC3B puncta formation. In HeLa cells (Figure 6A), treatment with CBD and THC led to a noticeable increase in LC3B puncta compared to the control. Cisplatin caused a moderate increase, while treatments with THC–CBD–cisplatin combination resulted in a significant accumulation of LC3B puncta, which may reflect autophagic activity. The positive control (Rapamycin) exhibited the highest induction of autophagy. In SiHa cells (Figure 6B), a similar pattern emerged, with THC and THC–CBD–cisplatin treatments producing marked LC3B puncta formation compared to single agents. In contrast, MCF‐12A non‐malignant cells (Figure 6C) displayed no LC3B puncta across treatments, with autophagy observed only in the positive control. Quantification of LC3B puncta per cell confirmed significant increases in autophagic activity in HeLa and SiHa cells under cannabinoid and combination treatments, with negligible effects in MCF‐12A cells (Figure 6D–F).

### 3.7 DNA Repair Genes

**FIGURE 7:**
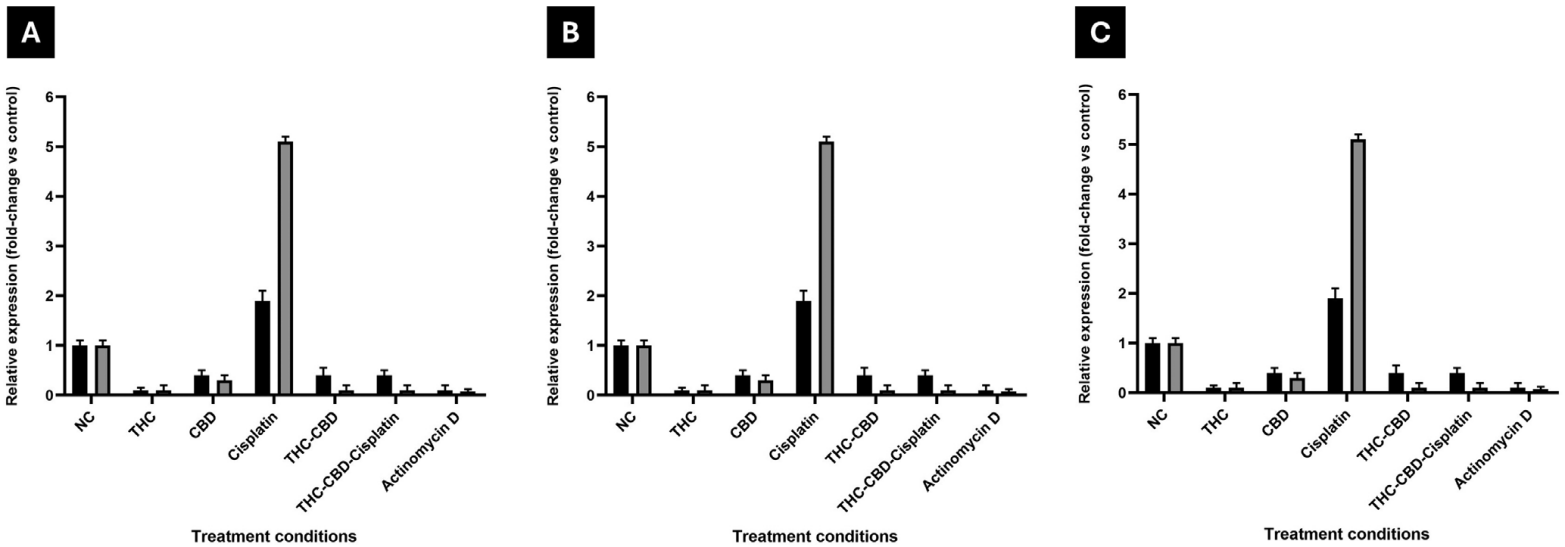
Expression of DNA repair genes *(XRCC1 and RAD51*) in (A) HeLa, (B) SiHa and (C) MCF‐12A cells after 72 h of treatment. The expression of *XRCC1* and *RAD51* was measured and is presented as fold change relative to the negative control (NC). A relative expression value > 1 indicates upregulation, while a value < 1 indicates downregulation. Standard deviation represented by T‐bars. Actinomycin D was used as a positive control.

Quantitative PCR analysis demonstrated that cisplatin alone significantly increased the expression of *XRCC1* and *RAD51* in HeLa cells by 2.3‐fold and 2.6‐fold, respectively (Figure 7A), and in SiHa cells by 2.1‐fold and 2.4‐fold (Figure 7B), respectively, indicating the activation of DNA repair pathways in response to genotoxic stress. In contrast, treatment with either THC or CBD alone resulted in a moderate reduction in the expression of both genes, with *XRCC1* decreasing to 0.8‐fold and 0.7‐fold, and *RAD51* to 0.6‐fold and 0.5‐fold in HeLa and SiHa cells, respectively. The combination of THC and CBD further reduced expression levels, with *XRCC1* at 0.5‐fold and *RAD51* at 0.4‐fold in HeLa cells, and *XRCC1* at 0.6‐fold and *RAD51* at 0.5‐fold in SiHa cells. The most significant reduction was observed with the THC–CBD–cisplatin combination, where *XRCC1* and *RAD51* levels dropped to 0.3‐fold and 0.2‐fold in HeLa cells, and 0.4‐fold and 0.3‐fold in SiHa cells. In non‐tumorigenic MCF‐12A cells, the expression of both genes remained near baseline (1.0‐fold), indicating minimal genomic stress (Figure 7C). These findings illustrate that the THC–CBD–cisplatin combination selectively reduces DNA repair gene expression in cancer cells, thereby enhancing cisplatin‐induced DNA damage and apoptosis.

## 4 Discussion

Cisplatin remains the gold standard chemotherapeutic agent for treating several solid tumors, including cervical cancer [4]. However, its clinical application is limited by severe systemic toxicity and the development of chemoresistance [17]. The aim of the study was to assess the effect of the combination of cisplatin with phytocannabinoids, THC and CBD, on cell proliferation, morphology, cell cycle progression, cell death, and DNA damage. It was found that cannabinoid co‐treatment amplified the anti‐proliferative and pro‐apoptotic effects of cisplatin in HeLa and SiHa cells, while having minimal impact on non‐cancerous MCF‐12A cells. Although apoptotic morphology was observed in MCF‐12A cells, the response was significantly less pronounced than that in cancer cell lines, supporting the relative selectivity of the triple combination. The observed antagonism of the triple combination in MCF‐12A cells suggests that this regimen could potentially reduce the systemic side effects typically associated with high‐dose cisplatin alone. These results support the growing evidence that cannabinoids can serve as selective adjuvants, enhancing chemotherapeutic activity while reducing off‐target toxicity [18].

The marked decrease in proliferation and increase in apoptosis observed with the THC–CBD–cisplatin combination is consistent with reports that cannabinoids induce apoptosis through mitochondrial dysfunction [19, 20], reactive oxygen species generation [21], and caspase activation [22]. In a recent literature it was noted that THC and CBD upregulate pro‐apoptotic proteins while downregulating the anti‐apoptotic Bcl‐2 in cervical cancer cells, resulting in caspase‐dependent cell death [10]. The synergistic effect observed in this study supports previous evidence that cannabinoids sensitize tumor cells to chemotherapeutic stress by amplifying oxidative and endoplasmic reticulum stress [23]. This mechanistic convergence likely explains the enhanced cytotoxicity of the triple‐combination treatment compared to individual agents.

The immunofluorescence analysis (Figure 6) revealed an increase in LC3B puncta formation in HeLa and SiHa cells following exposure to THC and CBD, indicating that there may be activation of autophagy. This finding is consistent with evidence that cannabinoids induce autophagy by inhibiting the AKT/mTOR pathway and upregulating ER‐stress markers p8 and TRIB3 [2, 24]. Notably, a more pronounced increase was observed with the THC‐CBD combination compared to THC–CBD–cisplatin. While THC–CBD alone significantly promoted LC3B puncta formation, the addition of cisplatin resulted in a reduced abundance of these puncta. This suggests a mixed response, where cisplatin directs some cells toward caspase‐dependent apoptosis and/or enhances autophagic flux and autolysosomal clearance, thereby decreasing the steady‐state accumulation of autophagosomes [25]. Although autophagy is often regarded as a survival mechanism, its sustained activation can lead to apoptosis under prolonged stress, particularly when combined with DNA‐damaging agents like cisplatin [26]. The minimal to no autophagic activity observed in MCF‐12A cells suggests that this response may be tumor‐selective.

At the molecular level, cisplatin monotherapy significantly upregulated *XRCC1* and *RAD51* (Figure 7), which are both essential for base‐excision and homologous recombination repair, respectively. This observation is consistent with previous studies, indicating that DNA repair is a primary adaptive response to cisplatin‐induced adduct formation [27]. However, when combined with cannabinoids, there was a notable downregulation of these genes, suggesting suppression of DNA repair capability. Similar effects have been observed in other cancers, where CBD reduced the expression of *BRCA1*, upregulation of *PARP1* in head and neck cancer cell lines [22], and *RAD51* [28], thereby sensitizing tumor cells to genotoxic stress. These findings suggest that cannabinoids enhance the efficacy of cisplatin by inhibiting the repair of DNA lesions, thus promoting apoptosis. This mechanistic synergy also provides a rationale for overcoming cisplatin resistance, which is a significant challenge in recurrent cervical cancer [29].

Cannabinoids have been shown to modulate resistance pathways by inhibiting drug‐efflux pumps like P‐glycoprotein, increasing intracellular ROS levels, and downregulating AKT survival signaling [10]. Together, these processes enhance tumor sensitivity to chemotherapy while maintaining the viability of normal cells. In the current study, the selective cytotoxicity observed in cancer cells, but not in MCF‐12A cells, supports this model. Cannabinoids preferentially target malignant cells through differential stress‐response modulation, confirming the tumor‐selective potential of such combinations [10]. This selectivity is clinically advantageous as it may allow for lower cisplatin dosing, thereby reducing nephrotoxicity and neurotoxicity.

The combination of cannabinoids and cisplatin aligns with emerging multimodal strategies that integrate chemotherapy, immunotherapy, and nanotechnology [10]. Delivering cannabinoid–cisplatin combinations via nanoparticles could potentially enhance bioavailability, tumor accumulation, and therapeutic efficacy while reducing systemic exposure [30]. Future research should explore whether encapsulating these combinations within lipid‐ or polymeric nanocarriers can replicate or enhance the synergistic effects observed in vitro.

This data collectively demonstrates that combining THC and CBD with cisplatin enhances the anti‐cancer effects in cervical cancer cells by inducing apoptosis and autophagy while suppressing DNA repair pathways. These effects were selective for malignant cells and align with mechanisms reported in recent literature [31]. The findings highlight the potential of cannabinoid‐based adjuvants to overcome cisplatin resistance and reduce chemotherapy‐related toxicity, warranting further exploration in preclinical and clinical models.

While the synergistic potential of THC and CBD in enhancing cisplatin's efficacy is promising, translating these in vitro findings to clinical applications necessitates addressing significant pharmacokinetic challenges inherent to these cannabinoids. Both THC and CBD are characterized by low and highly variable oral bioavailability, typically ranging from 6% to 20% due to their high lipophilicity and extensive first‐pass metabolism in the liver. Furthermore, their rapid metabolism, primarily via the cytochrome P450 enzyme system, may lead to fluctuations in plasma concentrations that could impact the consistency of the synergistic effects observed in this study [32]. Factors such as the tumor microenvironment and immune modulation can also influence drug responses in vivo [33]. Additionally, the psychoactive properties of THC and the variability in cannabinoid formulations present regulatory and dosage challenges [34]. Therefore, further in vivo validation and dose–response optimization are required. Future translational research must therefore consider advanced delivery systems, such as nanoparticle formulations, to optimize therapeutic delivery and ensure that effective concentrations reach the target cervical tumor tissues while minimizing systemic variability. Furthermore, this study evaluated effects in HeLa and SiHa cell lines, as these represent the most common clinical presentations of HPV‐positive cervical cancer. Future studies could be expanded to include CaSki and C33A cell lines.

## 5 Conclusion

This study demonstrates that the combination of cannabinoids, specifically THC and CBD, with cisplatin results in enhanced, selective, and mechanistically diverse anticancer effects in cervical cancer cells. The combined treatment induces apoptosis and autophagy while inhibiting DNA repair capacity, leading to significant cytotoxicity against cancer cells and minimizing damage to normal cells. These findings underscore the potential of cannabinoid‐based combination therapies as a promising and safer approach for cervical cancer treatment. The observed synergy between cannabinoids and cisplatin lays the groundwork for further preclinical development and eventually, clinical translation of more targeted and less toxic cancer therapies.

## Author Contributions

**S. P. Mathibela:** conceptualization, investigation, writing – original draft, validation, methodology, data curation, visualization, formal analysis. **M. T. Lebelo:** conceptualization, funding acquisition, methodology, validation, writing – review and editing, project administration, supervision, resources, formal analysis. **V. Steenkamp:** conceptualization, methodology, validation, writing – review and editing, software, formal analysis, project administration, supervision, resources.

## Funding

This work was supported by the Research Development Programme, University of Pretoria and the National Research Foundation (NGAP250520314378).

## Ethics Statement

The study was conducted according to the guidelines of the Declaration of Helsinki, and approved by the ethics committee of the University of Pretoria, Faculty of Health Sciences (207/2024).

## Conflicts of Interest

The authors declare no conflicts of interest.

## Data Availability

The data that support the findings of this study are available from the corresponding author upon reasonable request.
